# Acute Appendicitis: An Extracolonic Manifestation of* Clostridium difficile* Colitis

**DOI:** 10.1155/2017/5083535

**Published:** 2017-07-03

**Authors:** Ali Ridha, Shoaib M. Safiullah, Sarah Al-Abayechi, Amin Ur Rehman Nadeem

**Affiliations:** ^1^University of Arkansas for Medical Science, 4301 West Markham Street, Little Rock, AR 72205, USA; ^2^Chicago Medical School, Rosalind Franklin University of Medicine and Science, 3333 Green Bay Rd, North Chicago, IL 60064, USA

## Abstract

The current report is the case of a 30-year-old male patient who presented with symptomatology suggestive of appendicitis. However, careful history-taking and laboratory tests led to the diagnosis of* Clostridium difficile* colitis, resulting in successful nonsurgical management of this patient. Although both appendicitis and* C. difficile *colitis are common conditions, they are rarely diagnosed concurrently. This is reflected by paucity of literature describing this manifestation. Given this current presentation, the authors contend that the manifestation of extracolonic colitis within the appendix is possibly underdiagnosed or misdiagnosed as an acute appendicitis and thus potentially results in unnecessary surgical intervention. This report reminds physicians to consider the medical approach to managing acute appendicitis given the possibility of underlying* C. difficile *colitis as the causative factor.

## 1. Introduction

Acute appendicitis is considered among the most common abdominal surgical emergencies, a condition with an etiology that has yet to be elucidated and is currently considered multifactorial [1, 2].* C. difficile *colitis is another common healthcare-associated condition and is a significant cause of morbidity and mortality among older adult hospitalized patients [3]. Appendicitis rarely presents secondary to colitis; indeed, to our knowledge, only three cases of appendicitis induced by* C. difficile *infection have been reported in the literature [4–6].

## 2. Case Report

A 30-year-old Hispanic man presented to the emergency department with several episodes of vomiting and nonbloody diarrhea for two days. This was accompanied by generalized abdominal cramps and dizziness. There was no history of recent travel, sick contacts, similar past illness, new foods, or medication changes. He denied fever, chest pain, cough, headache, shortness of breath, palpitations, loss of consciousness, or urinary complaints. Upon further questioning, the patient stated he had taken clindamycin for cellulitis two months priorly. Past medical and family history were noncontributory.

At the emergency department, patient was alert and oriented to person, place, and time and in no acute distress. He was afebrile, blood pressure was 79/50 mm of Hg, and heart rate was 108/minute. Cardiac, pulmonary, and skin examinations were normal. Abdominal examination revealed vague, mild, and diffuse abdominal tenderness on palpation. Bowel sounds were hyperactive and rectal tone was intact. The remainder of his physical examination was negative for other sources of infection.

Initial blood workup included complete blood count and revealed a white blood cell count of 19.2 × 10^9^/L, hemoglobin 14.4 g/dL, hematocrit 41.2%, and platelets 90 × 10^9^/L. A metabolic panel showed BUN 43 mg/dL, creatinine 5.12 mg/dL, AST 103 U/L, ALT 227 U/L, total bilirubin 3.2 mg/dL, and lactate 2.6 mg/dL. Coagulation profile revealed INR 1.2, PTT 37.9 seconds, and PT 14.3 seconds. Blood smear did not reveal schistocytes or fragmented RBCs. He had a normal haptoglobin, LDH, reticulocyte index, and fibrinogen. A sample for stool studies was sent at that time.

**Figure 1:**
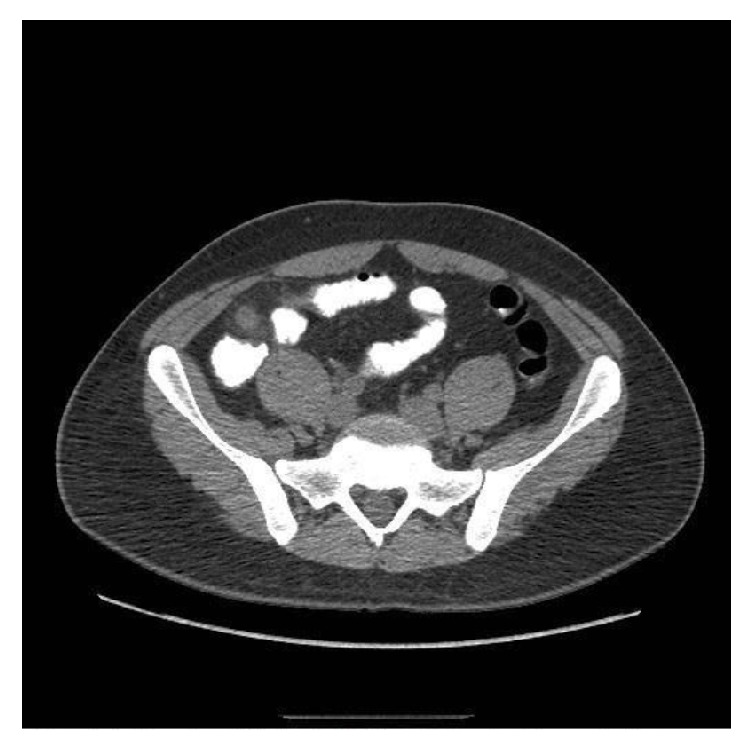
Axial section of abdominal CT scan with contrast demonstrating swollen appendix with 9.8 mm diameter, no appendicolith present.

**Figure 2:**
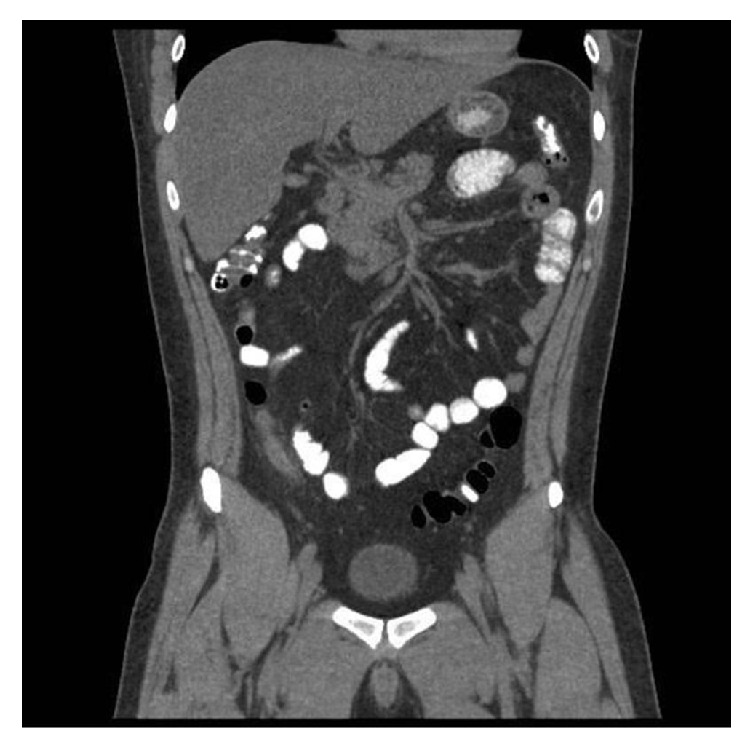
Coronal section of abdominal CT scan with contrast demonstrating swollen appendix with 9.8 mm diameter, no appendicolith present.

Given clinical and laboratory findings suggestive of sepsis, the patient was started on intravenous normal saline, metronidazole, and levofloxacin. He was then admitted under our care to the intensive care unit on that day for close monitoring; we made no subsequent changes in his management. An abdominal computed tomography indicated an enlarged appendix which measured 9.8 mm with periappendiceal inflammation suggestive of acute appendicitis without abscess (Figures 1 and 2). The general surgery service was consulted, who then recommended surgical intervention. As the patient was being prepared for surgery, his stool study polymerase chain reaction results returned as positive for* Clostridium difficile* toxin (NAP1 strain). The surgical service, therefore, decided that the patient should be continued on medical management, with plans to operate if a localized peritonitis were to develop in this setting. We subsequently obtained a consultation from our infectious disease specialists who added oral vancomycin to the patient's regimen.

The remaining stool studies were negative for ova, parasites,* Salmonella*,* Shigella*,* Campylobacter jejuni*, and* Escherichia coli*. Workup for other sources of infections, including chest X-ray and blood and urine cultures, were negative. On the following day, the patient's laboratory studies showed significant improvement, and he appeared clinically better. Thus, the patient was transferred to the general medical floor for continued management. He was discharged home, following three days of observation, with instructions to continue oral vancomycin for 6 additional days and follow up with his primary care physician in one week.

## 3. Discussion

Although* Clostridium difficile* colitis and appendicitis are each very common independently,* C. difficile* as an etiology of appendicitis is uncommon. There have been only three reported cases of appendicitis induced* by C. difficile* infection in the literature [4–6]. Secretory diarrhea and* C. difficile* colitis are the most common complications for* C. difficile* toxins A and B after administration of any antimicrobial drug, but especially with ampicillin, cephalosporins, fluoroquinolones, and clindamycin [7]. Reports of extracolonic manifestations of* C. difficile* include bacteremia, visceral abscess formation (splenic abscess, pancreatic abscess, empyema, and pleural effusion), prosthetic device infections, encephalopathy, reactive arthritis, osteomyelitis, and soft tissue infections [8, 9].

The current report is a very rare case of acute appendicitis as an extracolonic manifestation of* C. difficile*. Although surgical treatment is often sought for appendicitis, studies have suggested medical treatment may be sufficient (albeit with possibility of recurrence). Moreover, if the etiology of the appendicitis is a treatable infection as in our case, it warrants the consideration of a medical approach; surgery could be scheduled later when the patient is stable. We present this report as a reminder to our physician colleagues to consider* C. difficile* colitis in their differential diagnosis in a similar clinical scenario.
